# The SWiFT trial (Study of Whole Blood in Frontline Trauma)—the clinical and cost effectiveness of pre-hospital whole blood versus standard care in patients with life-threatening traumatic haemorrhage: study protocol for a multi-centre randomised controlled trial

**DOI:** 10.1186/s13063-023-07711-4

**Published:** 2023-11-14

**Authors:** Jason E. Smith, Ed B. G. Barnard, Charlie Brown-O’Sullivan, Rebecca Cardigan, Jane Davies, Annie Hawton, Emma Laing, Joanne Lucas, Richard Lyon, Gavin D. Perkins, Laura Smith, Simon J. Stanworth, Anne Weaver, Tom Woolley, Laura Green

**Affiliations:** 1 Academic Department of Military Emergency Medicine, Royal Centre for Defence Medicine, Birmingham, UK; 2 University Hospitals Plymouth NHS Trust, Plymouth, UK; 3 Cambridge University Hospitals NHS Foundation Trust, Cambridge, UK; 4 NHS Blood and Transplant Clinical Trials Unit, Cambridge, UK; 5 NHS Blood & Transplant, Bristol, UK; 6 Department of Haematology, University of Cambridge, Cambridge, UK; 7 Health Economics Group, University of Exeter, Exeter, UK; 8 Intensive Care National Audit and Research Centre (ICNARC), London, UK; 9 Air Ambulance Kent Surrey Sussex, Rochester, UK; 10 Department of Health Sciences, University of Surrey, Guildford, UK; 11 Warwick Medical School, University of Warwick, Coventry, CV4 7AL UK; 12 Oxford University Hospitals, Oxford, UK; 13 University of Oxford, Oxford, UK; 14 London’s Air Ambulance and Royal London Hospital, London, UK; 15 Academic Department of Military Anaesthesia and Critical Care, Royal Centre for Defence Medicine, Birmingham, UK; 16 Barts Health NHS Trust, London, UK; 17 Queen Mary University of London, London, UK

**Keywords:** Emergency medicine, Major trauma, Major haemorrhage, Transfusion, Pre-hospital, Whole blood

## Abstract

**Background:**

Early blood transfusion improves survival in patients with life-threatening bleeding, but the optimal transfusion strategy in the pre-hospital setting has yet to be established. Although there is some evidence of benefit with the use of whole blood, there have been no randomised controlled trials exploring the clinical and cost effectiveness of pre-hospital administration of whole blood versus component therapy for trauma patients with life-threatening bleeding. The aim of this trial is to determine whether pre-hospital leukocyte-depleted whole blood transfusion is better than standard care (blood component transfusion) in reducing the proportion of participants who experience death or massive transfusion at 24 h.

**Methods:**

This is a multi-centre, superiority, open-label, randomised controlled trial with internal pilot and within-trial cost-effectiveness analysis. Patients of any age will be eligible if they have suffered major traumatic haemorrhage and are attended by a participating air ambulance service. The primary outcome is the proportion of participants with traumatic haemorrhage who have died (all-cause mortality) or received massive transfusion in the first 24 h from randomisation. A number of secondary clinical, process, and safety endpoints will be collected and analysed. Cost (provision of whole blood, hospital, health, and wider care resource use) and outcome data will be synthesised to present incremental cost-effectiveness ratios for the trial primary outcome and cost per quality-adjusted life year at 90 days after injury. We plan to recruit 848 participants (a two-sided test with 85% power, 5% type I error, 1-1 allocation, and one interim analysis would require 602 participants—after allowing for 25% of participants in traumatic cardiac arrest and an additional 5% drop out, the sample size is 848).

**Discussion:**

The SWiFT trial will recruit 848 participants across at least ten air ambulances services in the UK. It will investigate the clinical and cost-effectiveness of whole blood transfusion versus component therapy in the management of patients with life-threatening bleeding in the pre-hospital setting.

**Trial registration:**

ISRCTN: 23657907; EudraCT: 2021-006876-18; IRAS Number: 300414; REC: 22/SC/0072, 21 Dec 2021.

## Administrative information

Note: the numbers in curly brackets in this protocol refer to SPIRIT checklist item numbers. The order of the items has been modified to group similar items (see http://www.equator-network.org/reporting-guidelines/spirit-2013-statement-defining-standard-protocol-items-for-clinical-trials/).

**Table:** 

Title {1}	SWiFT (Study of Whole blood in Frontline Trauma): a Multi-Centre Randomised Controlled Trial of the Clinical and Cost Effectiveness of Pre-Hospital Whole Blood Administration versus Standard Care for Traumatic Haemorrhage
Trial registration {2a and 2b}	ISRCTN: 23657907; EudraCT: 2021-006876-18
Protocol version {3}	V1.1 dated 03/05/2022
Funding {4}	Funding was secured from NHS Blood and Transplant (NHSBT), the UK Ministry of Defence and participating Air Ambulance Charities. Although employees of NHSBT, the MOD and participating Air Ambulances are involved in the conduct of the study, the funding bodies themselves had no role in the design of the study; collection, analysis, and interpretation of data or in writing this manuscript.
Author details {5a}	Jason E Smith Academic Department of Military Emergency Medicine, Royal Centre for Defence Medicine, Birmingham, and University Hospitals Plymouth NHS Trust, Plymouth, UK. jasonesmith@nhs.net Ed B G Barnard, Academic Department of Military Emergency Medicine, Royal Centre for Defence Medicine, Birmingham, and Cambridge University Hospitals NHS Foundation Trust, Cambridge, UK. edward.barnard@nhs.net Charlie Brown-O’Sullivan, NHS Blood and Transplant Clinical Trials Unit charlie.brown@nhsbt.nhs.uk Rebecca Cardigan, NHS Blood & Transplant and Department of Haematology, University of Cambridge, UK rebecca.cardigan@nhsbt.nhs.uk Jane Davies, NHS Blood & Transplant Jane.Davies@nhsbt.nhs.uk Annie Hawton, Health Economics Group, University of Exeter A.Hawton@exeter.ac.uk Emma Laing, Intensive Care National Audit and Research Centre (ICNARC) emma.laing@icnarc.org Joanne Lucas, NHS Blood and Transplant Clinical Trials Unit joanne.lucas@nhsbt.nhs.uk Richard Lyon, Air Ambulance Kent Surrey Sussex; Department of Health Sciences, University of Surrey RichardLyon@aakss.org.uk Gavin D Perkins, Warwick Medical School, University of Warwick, Coventry, CV4 7AL, UK g.d.perkins@warwick.ac.uk Laura Smith, NHS Blood and Transplant Clinical Trials Unit Laura.Smith2@nhsbt.nhs.uk Simon J Stanworth, NHS Blood & Transplant, Oxford University Hospitals, and University of Oxford simon.stanworth@nhsbt.nhs.uk Anne Weaver, London’s Air Ambulance and Royal London Hospital anne.weaver@nhs.net Tom Woolley, Academic Department of Military Anaesthesia and Critical Care, Royal Centre for Defence Medicine, Birmingham, UK t.woolley@nhs.net Laura Green, NHS Blood & Transplant, Barts Health NHS Trust, and Queen Mary University of London, UK Laura.Green@nhsbt.nhs.uk

## Introduction

### Background and rationale {6a}

Major trauma kills more than 5400 people every year in the UK [1] and globally more than human immunodeficiency virus-acquired immunodeficiency syndrome (HIV-AIDS), tuberculosis (TB), and malaria combined [2]. Uncontrolled bleeding accounts for a significant proportion of these deaths, with approximately 20% occurring in the first 24 h and 40% occurring within the first 30 days [3, 4]. Blood transfusion is a life-saving treatment in the management of bleeding until it is controlled in hospital by surgery or interventional radiology. Blood transfusion is delivered through different blood components, namely red blood cells (RBC), plasma, and platelets; all these components are derived from whole blood (WB) donation and are stored separately at different temperatures.

Observational studies in military [5] and civilian [6] settings have reported a 12–14% absolute reduction in 30-day mortality with pre-hospital RBC transfusion, with the effect being greater if transfusion is started within 15 min of medical evacuation [5]. However, recent trials investigating the use of pre-hospital blood transfusion have shown conflicting results. The RePHILL trial showed no benefit in a composite primary outcome of lactate clearance and episode mortality from transfusion with RBC plus lyophilised plasma when compared to resuscitation with normal saline [3]. Two US trials investigating the use of pre-hospital plasma resuscitation also showed conflicting results [7, 8], although combined data from these trials suggest that there is survival benefit, which is greatest in patients who received both RBC and plasma [9], in patients with blunt trauma [10], and when pre-hospital times are longer (than 20 min) [11].

In the UK, many patients who are bleeding at the scene of an incident are transfused blood before they arrive at hospital. This is most commonly a combination of RBC and plasma [4]. In a national survey in 2020, of the 18 UK Air Ambulance Services that responded, 67% administered red blood cells (RBC) and plasma, 22% administered RBC only, and 11% plasma only [12]. In this survey, over 80% stated that WB would be their preferred component, but the evidence for its use is limited.

Systematic reviews, comparing the impact of WB transfusion versus blood component therapy on 24-h and 30-day mortality for adult trauma patients with acute major haemorrhage, showed no clear benefit with WB transfusion [13–15]. In these reviews, there was only one randomised controlled trial (*n* = 107 patients), which was not powered to demonstrate a difference in survival. Since the publication of these systematic reviews, an observational study in the USA comparing WB to component therapy (RBC and or plasma) reported a twofold increase in likelihood of 30-day survival with WB transfusion (odds ratio, 2.19; 95% CI 1.01–4.76; *p* = 0.047) [16].

The optimal blood transfusion strategy in the pre-hospital setting has yet to be established. The implementation of WB into routine practice would result in significant changes to the current manufacturing processes for the blood services and potentially increased cost. Although there is some evidence of benefit with the use of WB, there have been no prospective randomised controlled trials exploring the clinical and cost effectiveness of pre-hospital administration of WB versus component therapy for bleeding trauma patients. It is therefore essential for patients, healthcare professionals and blood services that the clinical and cost effectiveness of pre-hospital WB transfusion is evaluated in a large trial before its widespread implementation in the NHS.

### Objectives {7}

The primary objective is to determine whether pre-hospital leukocyte-depleted whole blood transfusion is better than standard care (component transfusion) in reducing the proportion of participants who experience death or massive transfusion at 24 h. The secondary objectives are to determine all-cause mortality at 6 h, 24 h, 30 days, and 90 days; morbidity up to 30 days (number of organ failure-free days, number of days in critical care and in hospital); hospital resource use up to 30 days, discharge, or death, including organ failure-free days, time spent in critical care and total in-patient stay, blood components, and additional haemostatic agents received; cost-effectiveness; and safety.

### Trial design {8}

The trial design is a multi-centre, pragmatic, superiority, randomised controlled, open-label, parallel group, two-arm trial with internal pilot and within-trial cost-effectiveness analysis.

## Methods: participants, interventions, and outcomes

### Study setting {9}

Air Ambulance Services (AAS) are responsible for treating patients on scene and delivering the trial intervention prior to hospital admission. These sites will be Air Ambulance Services in the UK that deliver a combination of RBC and plasma as standard care for the treatment of life-threatening bleeding.

Transfusion laboratory sites are responsible for supplying blood components in accordance with the randomisation procedure. These sites are the hospitals (transfusion laboratories) that supply blood components to the participating Air Ambulance Services.

Receiving hospital sites are secondary care sites where participants will be admitted following pre-hospital trial treatment. These sites will be designated major trauma centres, trauma units, or other hospitals that receive patients from the participating Air Ambulance Services.

### Eligibility criteria {10}

The pragmatic nature of this trial means that the decision to administer the intervention will be based on clinician judgement and according to the AAS usual criteria for initiation of blood transfusion. As injured children and adults are routinely managed by Air Ambulance Services and may receive pre-hospital blood transfusion, patients of all ages will be included in the study. Inclusion criteria are therefore a patient (of any age) who has suffered a traumatic injury, is attended by a participating Air Ambulance Service clinical team, and who requires pre-hospital blood transfusion to treat major traumatic haemorrhage. Exclusion criteria are inability to achieve intravenous or intraosseous access; if it is known that a patient will object to being given blood transfusion for any reason; or if blood has already been administered on-scene, prior to arrival of the participating AAS.

### Who will take informed consent? {26a}

Participants will be enrolled under an emergency waiver of consent.

Major traumatic haemorrhage is a life-threatening condition that requires urgent treatment. The vast majority of participants will lack capacity and/or will be minors of unknown age. Due to the emergency nature of major trauma, time will not allow for written informed consent to be obtained prior to the intervention being administered. Therefore, it would be inappropriate to attempt to gain informed consent at this time, as it could delay life-saving treatment. It would also be clinically unjustifiable to delay treatment until full informed consent can be obtained from a personal legal representative or parent/guardian. Even if such a representative or person with parental responsibility were immediately available, the emotional distress of the situation is such that they would be unlikely to make an informed decision in the minimal time available.

Consequently, participants will be enrolled under an emergency waiver of consent. Participants who are incapable of giving consent in emergency situations are an established exception to the general rule of informed consent in clinical trials. This is acknowledged in the Declaration of Helsinki, 2013. Under UK law (The Medicines for Human Use (Clinical Trials: Amendment No. 2) Regulations 2006), minors or incapacitated adults in emergency settings can be entered into a trial before informed consent is obtained.

Contact with trial participants and/or their relatives/friends/person with parental responsibility to initiate the consent process will be made by the local research team at the receiving hospital as soon as practically possible after the initial emergency has passed. Details of the informed consent discussions will be recorded in the participant’s medical notes.

In exceptional circumstances, it may not be possible to obtain informed consent (either via the participant directly, or via a personal/professional legal representative) before a participant is discharged from hospital. If no contact with the participant is possible, we will use the data that has previously been collected under the provisions of Section 251 of the National Health Service Act 2006 (with approval from the Confidentiality Advisory Group (CAG)).

### Additional consent provisions for collection and use of participant data and biological specimens {26b}

Some receiving hospital sites are taking part in the Activation of Coagulation and Inflammation in Trauma 2 (ACIT-2) study, which involves collection of research blood samples after arrival at hospital. The arrangements for consent are described in the ACIT-2 study protocol.

## Interventions

### Explanation for the choice of comparators {6b}

The trial will compare up to two units of WB (intervention arm) versus up to two units of RBCs and two units of plasma (control arm). Following this, additional blood components will be administered as required (as per standard of care). The choice of control intervention was determined by the results of a national survey of air ambulances, conducted in 2020, which found that the majority of patients requiring pre-hospital transfusion in the UK receive a combination of RBCs and plasma [12].

### Intervention description {11a}

Eligible patients will receive either WB or a combination of RBC and plasma, delivered via an intravenous cannula or an intraosseous cannula, in the pre-hospital environment by the attending air ambulance service clinical team. If the transfusion is still in progress when the patient arrives at hospital, it can be continued until complete if it is still clinically indicated. Once complete, routine care will continue, including further blood products as per local protocol.

The WB component will be manufactured by NHS Blood and Transplant, which is the main blood supplier of blood in England. The WB units are derived from a single donor after the WB is collected into 66.5-mL citrate-phosphate-dextrose (CPD) anticoagulant and filtered to remove white cells [17, 18], as a variant Creutzfeldt–Jakob disease (CJD) safety measure step that is applied to all blood components manufactured in the UK since 1999.

Once manufactured, the WB units will be transported to hospitals under continuous temperature control as per standard operating procedures and stored in the transfusion laboratories at 2–6 °C for up to 21 days [17, 18]. The WB units will be group O, RhD negative, high titre negative for anti-A and anti-B, and Kell negative.

The control arm will consist of 2 units of RBC and 2 units of plasma (either thawed fresh frozen plasma, FFP, up to 5 days post-thawing or freeze-dried plasma (LyoPlas) [19, 20], dependent on the standard practice for each AAS. RBC and FFP are blood components that are supplied by NHS Blood and Transplant to the hospitals that are supporting this trial. LyoPlas is a freeze-dried plasma product derived from a single donation and is licenced for use in the same indication as FFP.

### Criteria for discontinuing or modifying allocated interventions {11b}

If the trial intervention has not been completed by the time the participant arrives in hospital, it should be completed after arrival at hospital if it is still clinically indicated. Further management is dictated by normal clinical protocols. If bleeding continues and further fluid resuscitation is needed, additional blood components will be administered as per standard care and as required, following the initial trial components. All blood components will be given according to local standard policies on the use of blood warmers.

### Strategies to improve adherence to interventions {11c}

The volume of blood product given will follow local protocol. For adult patients, this would usually be up to two units of WB or up to two units each of RBCs and plasma. For children, local protocol will be followed regarding the comparable number of units that should be received.

### Relevant concomitant care permitted or prohibited during the trial {11d}

WB, RBC, and LyoPlas are stored in citrate; therefore, solutions containing calcium will not be administered concurrently through the same cannula. Medicinal products should not be added to WB, RBC, thawed plasma, or LyoPlas. No other blood products (e.g. fibrinogen concentrate) should be given in the pre-hospital phase of care.

Due to the emergency nature of this trial, it is highly unlikely that those enrolling participants to SWiFT will be aware if a participant is already enrolled in a clinical trial. Where a SWiFT participant is subsequently found to have been participating in a concurrent trial or is being considered for enrolment into another trial, the site must notify the SWiFT CIs, who will in turn liaise with the CI for the other trial to determine whether co-enrolment is permitted.

### Provisions for post-trial care {30}

After the trial intervention is delivered, all further management will be according to standard local protocols. No specific provisions for post-trial care have been made.

### Outcomes {12}

The primary outcome is the proportion of participants who have died (all-cause mortality) or received massive transfusion in the first 24 h from randomisation. A participant is considered randomised and entered into the trial when the trial intervention box has been opened.

For the purposes of analysis, two units of whole blood will be counted as equivalent to four total units due to the volume difference between the intervention and control arm, the fact that two units of whole blood contain the equivalent of two units of plasma plus two units of packed RBCs, and for the massive transfusion part of the primary analysis, both arms will start will an equal baseline.

Secondary outcomes include the following: the proportion of participants who received massive transfusion (for adults, a total of 10 or more units of any blood components) in the first 24 h from randomisation; all-cause mortality at 6 h, 24 h, 30 days, and 90 days from randomisation; number of organ failure-free days up to 30 days, defined as the number of days free of advanced cardiovascular, advanced respiratory, and advanced renal support; days in critical care and separately in an acute care hospital (up to 90 days); units of each blood component received in the 24 h after randomisation, including pre-hospital transfusions (WB, RBC, plasma, platelets, and cryoprecipitate); amount of cell salvage received at 24 h (in ml) after randomisation; number of participants receiving additional haemostatic agents received at 24 h after randomisation (recombinant factor VIIa, fibrinogen concentrate, prothrombin complex concentrate (PCC), tranexamic acid (TXA)); presence of coagulopathy (defined as prothrombin time above the normal range) in the first sample taken on arrival at an acute care hospital; acid-base disturbance measured by lactate, base excess, and pH level in first sample taken on arrival at acute care hospital; cost of provision of the whole blood intervention; hospital resource use up to discharge or death, including ventilator days, days spent in critical care, and total in-patient stay; health, social, and wider care service resource use to 90 days after injury; health-related quality of life (EuroQol 5 Dimension 5 Level, EQ-5D-5L) at 90 days after injury; thrombosis (arterial and venous) up to 30 days after randomisation or hospital discharge or death, whichever is first; and all transfusion reactions/events relating to pre-hospital blood components which have been reported to SHOT (Serious Hazards of Transfusion) in the first 14 days after randomisation.

### Participant timeline {13}

**Fig. 1:**
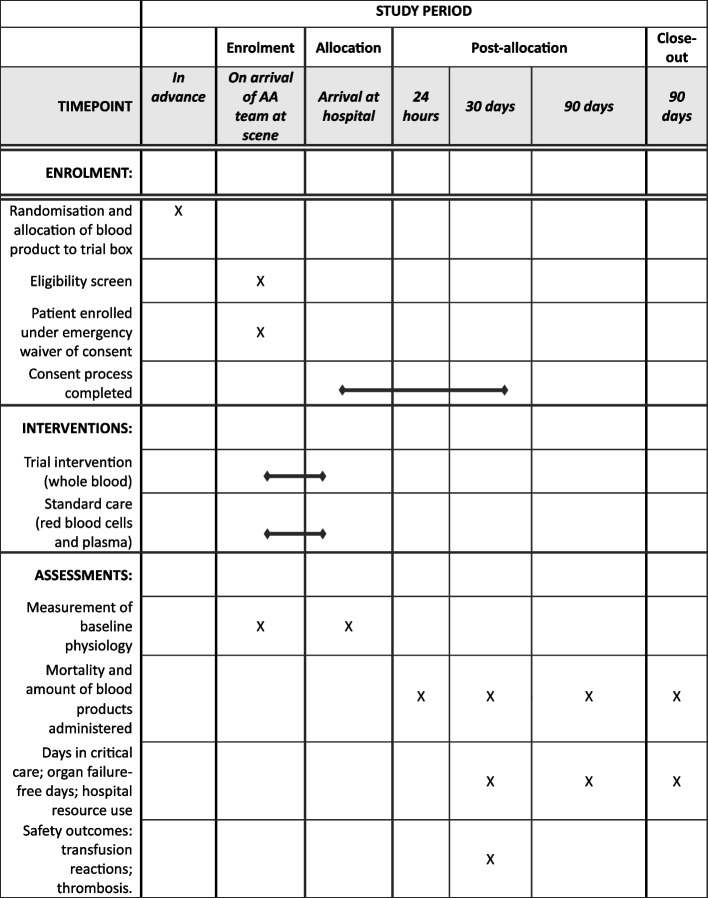
Schedule of enrolment, interventions, and assessments (AA, air ambulance)

The participant timeline is shown in Fig. 1.

### Sample size {14}

The trial will use a group sequential, superiority design and one interim analysis with O’Brien Fleming stopping boundaries, to inform early stopping for harm or benefit. Using data from the red cell and plasma (RCP) study [4], the composite endpoint of 24-h mortality or massive transfusion (defined as the total blood components given being greater than or equal to 10 units) in the first 24 h was calculated as 68%. We have therefore used a baseline event rate of 68% in our calculations.

Several studies have reported that WB transfusion versus blood components (or addition of platelets to RBC and plasma resuscitation) are likely to reduce mortality and overall transfusion, hence the reason for choosing this composite outcome. Williams [16] reported that a similar number of units were received in ED, although fewer units of RBC, plasma, and overall products after leaving ED (the median (IQR) were 0 (0–4) for WB and 3 (0–10) for traditional component therapy). Spinella [21] reported a 24-h mortality rate of 4% in WB vs 12% in component therapy (67% relative difference and 8% absolute difference). A higher chance of massive transfusion in WB compared to component therapy (89% vs 78%) though differences were expected as the groups were determined by the blood products transfused. A sub-study of the Pragmatic, Randomized Optimal Platelet and Plasma Ratios (PROPPR) trial [22] showed an 11% absolute (66% relative difference) in 24-h mortality between those patients who did and did not received platelets. Perkins [23] reported a significant reduction in blood product use at 24 h in the WB group. The RCT of WB versus components showed similar results, but only in a sub-group analysis of patients without severe brain injury (median [IQR] 24-h total transfusion was 11 units [5, 17] vs. 16 units [4,41], *p* = 0.02) [23].

Hence, combining all the evidence above, we have powered the study to detect a 12% absolute (38% relative) difference in the composite endpoint between the two treatment arms (68% vs 56%). A two-sided test with 85% power, 5% type I error, 1-1 allocation, and one interim analysis would require 602 participants. After allowing for 25% of participants being in traumatic cardiac arrest and an additional 5% drop out, the total number of participants required for this trial is 848.

### Recruitment {15}

A national survey of pre-hospital practice, conducted in 2020 [12], and the red cell and plasma study conducted in England [4] were key to estimating the number of patients per year receiving blood transfusion delivered by each participating air ambulance.

The air ambulances taking part in the study will carry a trial intervention box containing blood products (either WB or standard care), and when they identify a patient requiring pre-hospital blood transfusion, they will open the box and administer the blood products. This should ensure that if a patient requires a blood transfusion and is attended by a participating air ambulance service, they are recruited into the trial.

## Assignment of interventions: allocation

### Sequence generation {16a}

A restricted randomisation method will be used in this trial. It will consist of randomly permuted blocks of varying undisclosed sizes, and stratified by AAS, to account for variation in trauma care and type of trauma between delivery sites. There will be a 1:1 allocation ratio, to the intervention and control arms. The allocation sequence will be produced from a specification provided by the trial statistician to Sealed Envelope (a centralised web-based randomisation service) and quality checked by the trial statistician.

### Concealment mechanism {16b}

Allocation will be conducted by the participating transfusion laboratory teams using Sealed Envelope, a centralised web-based randomisation service. Only Sealed Envelope and the trial statisticians will have access to the randomisation list.

### Implementation {16c}

On randomisation, an email notification will be sent to the transfusion laboratory team and NHSBT CTU, which will include the randomisation number, date and time of randomisation, and the allocated treatment (whole blood or standard care). The randomisation number must be added to the trial box and will be used on all subsequent study documentation.

Using the randomised allocation, boxes of blood components will be prepared by the participating transfusion laboratory teams. Transfusion laboratories will be supplied with pre-printed ‘SWiFT randomisation number’ labels to which they will add by hand the randomisation number. A registered user at the transfusion laboratories will perform a unique randomisation for each box to be used by the AAS. The allocated trial intervention will be packed into transport boxes, ensuring that the randomisation number is clearly documented on the outside of the box. The packed, sealed transport boxes will be dispatched to the AAS using an established courier service as required for each site. Unused boxes should be returned to the transfusion laboratory and if unopened replaced “like-for-like”. A new randomisation will only be performed once the box has been used (i.e. contents have been transfused to a participant) or if the box has been returned opened but the contents were not used.

## Assignment of interventions: blinding

### Who will be blinded {17a}

The AAS clinical teams will be blinded to the randomised allocation until the trial box is opened, after which point it is not feasible to maintain blinding. To ensure that the team can remain blinded until the box is opened, the boxes containing intervention or control will be the same size and shape. It is not possible to blind the team at the receiving hospital to the intervention.

Participants will be blinded to allocation until the trial box is opened, but they may discover this information post-randomisation (e.g. by reading their medical notes or requesting this information from the Research Team), although there is no obligation to inform the participant which treatment they received.

### Procedure for unblinding if needed {17b}

Not applicable. Unblinding will effectively occur when the trial box is opened.

## Data collection and management

### Plans for assessment and collection of outcomes {18a}

On arrival at hospital, the AAS clinical team will hand over clinical responsibility for the participant to the receiving hospital team and complete the pre-hospital form (on the eCRF). Each unit of blood administered pre-hospital will be recorded on the eCRF, along with the donation number (G number) of the unit and the transfusion start time.

The principal investigator and research team at the receiving hospital will be responsible for collecting the clinical data for trial participants.

The transfusion laboratory at the receiving hospital will be responsible for recording the final ‘fate’ of all blood components that were administered to the participant, to ensure full traceability.

Data to be collected by the research team at 24 h post-randomisation include the following: participant demographics and baseline data, participant status (alive/dead) at 6 h and 24 h, total number of blood components transfused, cell salvage/autologous blood transfusion, and any haemostatic agents received.

Data to be collected by the research team at 30 days post-randomisation include participant status (alive/dead), total number of blood components transfused, critical care unit admission (yes/no), plus date/time of admission where applicable, hospital resource use (up to discharge from acute care hospital or death, whichever is first), thromboembolic events (clinically diagnosed), and any transfusion reactions which have been reported to SHOT (occurring in first 14 days post-randomisation).

For participants that are alive at 90 days post-randomisation, data collected will include participant status (alive/dead), health-related quality of life questionnaire (EQ-5D-5L, or EuroQol 5 Dimension Youth, EQ-5D-Y for participants aged 5–14 years), and health, social, and wider care service use questionnaire. Limited patient identifiable information will be collected to enable linkage to the Trauma Audit and Research Network (TARN) and Intensive Care National Audit and Research Centre (ICNARC) routinely collected data. This will prevent duplicate data collection and reduce the burden of data collection for hospital research teams.

### Plans to promote participant retention and complete follow-up {18b}

With the exception of cases where consent is withdrawn, there are no other specific circumstances where a participant should be withdrawn from the trial. If a participant is withdrawn, a withdrawal form will be completed which will detail the reason for withdrawal, including whether we are able to use the data already collected.

### Data management {19}

The principal investigator has overall responsibility for data collection at each site. Participant data will be entered onto the trial database, which was designed and will be administered by the NHSBT Clinical Trials Unit Data Management team using OpenClinica. The OpenClinica database will be used for electronic data capture (EDC) management and reporting on this trial. Training and instructions for completion of eCRFs will be given to each site during at site activation.

All case report forms will be electronic. The eCRFs will be completed directly onto the EDC system (i.e. the database).

The NHSBT CTU staff will be in regular contact with local site personnel to check on progress and to help with any queries that may arise. Incoming electronic forms will be checked for completeness, consistency, timelines, and compliance with the protocol.

Direct access to eCRF data will be granted to authorised representatives from the sponsor, NHSBT CTU, host institution, and the regulatory authorities to permit trial-related monitoring, audits, and inspections in line with participant consent.

### Confidentiality {27}

Direct access to eCRF data will only be granted to authorised representatives from the sponsor, NHSBT CTU, host institution, and the regulatory authorities to permit trial-related monitoring, audits, and inspections in line with participant consent. Each participant will be allocated an individual trial identification number.

### Plans for collection, laboratory evaluation, and storage of biological specimens for genetic or molecular analysis in this trial/future use {33}

SWiFT participants will be eligible for enrolment in the ACIT-2 study at sites that are recruiting patients to that study. The details of the collection and analysis of the samples taken will be according to the ACIT-2 study protocol.

## Statistical methods

### Statistical methods for primary and secondary outcomes {20a}

Analyses will be described in detail in a full statistical analysis plan (SAP). This section summarises the main points.

The following factors will be used to assess baseline comparability of the randomised groups:

- ◦ Age in years, reported as a median (IQR)
- ◦ Number and proportion of paediatric participants (defined as < 16 years)
- ◦ Sex (male or female)
- ◦ Type of injury (blunt or penetrating)
- ◦ Nature of traumatic injury (high or low energy transfer)
- ◦ Injury Severity Score, reported as a median (IQR), defined as the sum of the squares of the highest AIS grade in each of the three most severely injured areas. Note if the participant is dead upon arrival to hospital, then the ISS is not calculated and hence will be assumed to be 75 (the maximum score) [24].
- ◦ Systolic blood pressure (mm Hg), reported as a median (IQR)
- ◦ Heart rate (per minute), reported as a median (IQR)
- ◦ Glasgow Coma Scale, reported as a median (IQR). This score measures eye opening, verbal and motor response functions
- ◦ Age of blood in days, reported as a median (IQR). This will be determined using routinely collected NHSBT data.

A CONSORT diagram will be produced to show the flow of participants through the trial.

#### Primary outcome

The primary outcome will be determined as the proportion of participants who have either died (from all causes) or received 10 or more units of blood components, within the 24 h from randomisation (a participant is considered randomised and entered into the trial when the trial intervention box has been opened). The following will be included in the blood component count: RBC, platelets, whole blood, thawed plasma (or LyoPlas), and cryoprecipitate. In this trial, for the purposes of analysis, two units of whole blood will be counted as equivalent to four total units due to the volume difference between the intervention and control arm, the fact that two units of whole blood contain the equivalent of two units of plasma plus two units of packed RBCs, and for the massive transfusion part of the primary analysis, both arms will start will an equal baseline. The composite outcome will be analysed using a mixed logistic regression model with adjustment for AAS, fitted as a random effect. A superiority hypothesis will be tested and the results from the adjusted analysis will be considered the primary analysis.

All analyses will be performed on a modified intention to treat basis, to exclude participants who experience a traumatic cardiac arrest, but will include all other randomised patients on whom a value of the response variable has been obtained, including those randomised in error and regardless of the participants’ adherence to the protocol. The data will be presented and analysed according to the arm to which the participant was randomised, regardless of whether they received the randomised intervention or not.

The primary outcome will be replicated per protocol, which will exclude participants who experience a traumatic cardiac arrest, those who experience a protocol deviation, were randomised in error or were withdrawn from the trial. The data will be presented and analysed according to the arm to which the participant was randomised.

#### Secondary outcome analysis

All-cause mortality at 6 h, 24 h, 30 days, and 90 days, the proportion of participants who receive 10 or more units of any blood components, and presence of coagulopathy will each be analysed separately using the same model that is described for the primary outcome.

Organ failure-free days and days in critical care and an acute care hospital will be reported as a median and IQR, by treatment arm and overall.

Units of each blood component received in the 24 h after randomisation will be summarised with a median and interquartile range and analysed using a negative binomial model, with adjustment for AAS.

Amount of millilitres (ml) of cell salvage at 24 h after randomisation and lactate, base excess, and pH level in first sample taken on arrival at acute care hospital will be analysed separately using a mixed linear regression model, with adjustment for AAS.

For each of the additional haemostatic agents, the number of participants who received each agent will be presented by trial arm and overall.

Thrombosis (arterial or venous thrombosis) up to 30 days after randomisation (or death or hospital discharge, whichever is first) and transfusion reactions/events relating to pre-hospital blood components which have been reported to SHOT (Serious Hazards of Transfusion) occurring in the first 14 days after randomisation will be summarised.

The economic evaluation will establish the resources required to provide the whole blood intervention, estimate intervention as compared to standard care costs, and conduct a full incremental cost-effectiveness analysis. Analyses will be undertaken against a primary perspective of the NHS/Social Care. Cost and outcome data will be synthesised to present incremental cost-effectiveness ratios for the primary outcome and cost per quality-adjusted life-year at 90 days. Multivariable regression analyses and bootstrapping will be employed, uncertainty will be explored, and cost-effectiveness acceptability curves will be presented. The internationally recognised CHEERs guidelines will be followed, and planned analyses will be described in a health economics analysis plan, which will be fully concordant with the SAP.

### Interim analyses {21b}

An interim analysis will be conducted after 400 participants, who did not experience traumatic cardiac arrest, have been randomised to inform early stopping of the trial in the case of strong evidence of harm or benefit.

A blinded analysis after the first 300 participants have been randomised and reached 24 h will allow us to reassess sample size requirements and recruitment rates, including estimating the underlying overall event rate, the dropout rates, and proportion of participants in traumatic cardiac arrest [25].

### Methods for additional analyses (e.g. subgroup analyses) {20b}

The primary outcome analysis will be replicated for four subgroups:

1. Presence of a traumatic brain injury defined as Abbreviated Injury Scale head score 3 or above
2. Adult vs paediatric (< 16 years)
3. Blunt vs penetrating traumatic injury
4. Injury Severity Score: ≤ 15 vs 16–25 vs ≥ 26

The last two of these subgroups have been added to allow comparison to previous trials.

We are interested in how the following factors are associated with the primary outcome. However, as these factors occur after randomisation and are not baseline characteristics, they cannot be subgroup analyses. Instead, each factor will be presented by treatment arm for the primary outcome. Then, each factor will be separately fitted in the primary outcome model to assess if the factor is significantly associated with the primary outcome after the other risk adjustment and how it affects the treatment effect. For the age of whole blood, only those in the intervention arm will be included, and hence, the data will just be tabulated by level of the factor against the primary outcome, and the treatment effect will not be explored.

1. Anaesthetised pre-hospital or not
2. Transport time to hospital: ≤ 20 min vs > 20 min
3. Age of whole blood: age of units of whole blood in days categorized into young (1–14 days) and old (> 14 days)

### Methods in analysis to handle protocol non-adherence and any statistical methods to handle missing data {20c}

Protocol deviations will be monitored throughout the trial and will be assessed as minor or major and whether they influence the statistical analysis. For the primary analysis, any missing data for the primary and secondary outcome measures will be treated as missing data and not be imputed. If an outcome has data missing for more than 25% of participants, then the analysis will not be undertaken. All missing primary and secondary outcome data will be summarised.

A sensitivity analysis will be conducted if the primary outcome is missing for more than 5% of participants, which will impute the composite endpoint.

### Plans to give access to the full protocol, participant-level data, and statistical code {31c}

The full trial protocol is available on request. The final dataset will reside with NHSBT. Access to the final dataset for additional analyses will be permitted under the agreement of the trial review committee (made up of the sponsor representative, chief investigators, trial statistician, and NHSBT information governance) and according to the trial publication policy.

## Oversight and monitoring

### Composition of the coordinating centre and trial steering committee {5d}

The trial will be managed by the NHSBT Clinical Trials Unit, with day-to-day input from a trial manager, trial coordinator, and clinical operations manager. A trial management group (TMG), made up of the two chief investigators, co-applicants, trial statisticians, and trial managers, will meet every 2 weeks during the trial set-up phase, and monthly thereafter, to monitor trial conduct, safety, recruitment, and follow-up.

An independent trial steering committee (TSC) has been convened with an independent chair, independent members with relevant clinical experience, and patient and lay representatives. The role of the TSC is to safeguard the well-being of patients and monitor overall conduct of this trial, e.g. organisation and implementation of the trial protocol. It should also provide advice through its independent chair to the trial management group (TMG), trial sponsor, and the CTU on all aspects of the trial. The TSC will be responsible for making executive decisions about the trial, considering advice and recommendations provided by the data monitoring committee (DMC).

### Composition of the data monitoring committee, its role and reporting structure {21a}

An independent data monitoring committee (DMC) has been convened with an independent chair and independent statistician. The DMC members will be the only individuals, along with the trial statisticians, to see outcome data by arm and overall while the trial is ongoing. It will review the trial progress and data management and make recommendations to the TSC and TMG. The DMC charter is stored in the Trial Master File at the CTU.

### Adverse event reporting and harms {22}

SWiFT trial participants have suffered traumatic injury and by definition will have multiple (serious) adverse events during their transfer to hospital and admission (related to their traumatic injury). Most of the AEs occurring in SWiFT, whether serious or not, will therefore be anticipated in the sense that they are recognised and accepted complications/consequences of major trauma.

All serious adverse events/reactions which relate to the administration of the pre-hospital WB, standard of care components (RBC and FFP), or LyoPlas will be reported as serious adverse events.

Any unused units will be disposed of in accordance with local requirements. For used products, all hospital transfusion laboratories are required by BSQR to keep the evidence of traceability for every unit used (or wasted) for 30 years. The following data items will be electronically recorded: donation number, component type, blood establishment which provided the blood component, date provided, identity of patient who received the blood component, or final fate if not transfused. Furthermore, transfusion laboratories have SOPs in place that accurately report any serious adverse events or reactions relating to blood transfusion to national haemovigilance systems (SHOT).

### Frequency and plans for auditing trial conduct {23}

The frequency, type, and intensity for routine monitoring and the requirements for “for cause” monitoring is detailed in the trial monitoring plan. The intensity of the monitoring according to the risk assessment is moderate (i.e. 1 visit/call per annum).

Most data can be monitored centrally to support trial and data integrity, safeguard patient safety, and monitor primary study endpoints. Therefore, one monitoring visit/call per year will be carried out via telephone or video conferencing, and key data points will be monitored centrally on a bimonthly basis.

In addition to potential Good Clinical Practice (GCP) inspections or audits by the host R&D department, the sponsor and NHSBT CTU reserve the right to conduct site audits, either as part of its ongoing audit programme or in response to adverse observations.

### Plans for communicating important protocol amendments to relevant parties (e.g. trial participants, ethical committees) {25}

Any amendments to the approved protocol will be communicated to all relevant parties in an expedited manner by the CTU via email.

### Dissemination plans {31a}

On completion of the trial, the data will be analysed and tabulated, and a final trial report prepared. The main trial results will be presented at national and international conferences and published in a peer-reviewed journal, on behalf of all collaborators. All presentations and publications related to the trial will be authorised by the TMG, and responsibility for all trial publications will rest with the TMG. The final report’s abstract and references will be made accessible, and participants will be able to access the results through the SWiFT trial website.

## Discussion

SWiFT is a pragmatic, randomised controlled trial that will determine if WB transfusion is superior to current standard of care (RBC and plasma) in the pre-hospital setting, in terms of reducing the 24-h mortality or requirement for massive transfusion. Furthermore, the trial has an integrated health economic evaluation to determine if WB is a cost-effective treatment, something not previously reported.

Over the last decade, interest in the use of WB in the management of traumatic haemorrhage has increased. NHS Blood and Transplant (NHSBT), the main blood supplier for hospitals in England, has demonstrated that WB with a shelf-life of 21 days can be manufactured for future use in the NHS. The implementation of WB into routine practice would result in significant changes to the current manufacturing processes for the blood services, as special leukocyte depletion filters are required to preserve the platelets within the WB. Furthermore, donors required to support WB transfusion for pre-hospital patients would need to be of a specific type (male, group O, RhD negative, and have low titre for anti-A and B antibodies in plasma), since the recipient blood group is not known in advance of giving the transfusion. These donors are a precious resource and are required to support other groups of patients (e.g. haemoglobinopathy); it is therefore important that this resource is utilised optimally. However, it could also be argued that early transfusion of WB may reduce the need for further blood component transfusion when patients arrive at hospital, due to earlier control of bleeding. The proposed trial would enable us to evaluate all these uncertainties.

The choice of the comparator arm represents current pre-hospital transfusion practice in England. Therefore, the main differences between the intervention and standard care arms are the addition of platelets in WB, and the fact that the WB transfusion is logistically easier and faster to administer than component therapy. We are not aware of any trials that have assessed the addition of platelets to RBC and/or plasma resuscitation in the pre-hospital setting, thus making this trial unique. A sub-study of the PROPRR trial in the hospital setting suggested that trauma patients who received early platelets had an 11% reduction in 24 h and 30-day mortality [22]. In the case of WB, platelets are cold-stored (unlike routine platelets that are stored at room temperature), and in vitro data have shown that platelet count declines in the WB during storage; however, platelets become more activated [18, 24, 26]. Therefore, it could be argued that activated platelets could be more effective in promoting haemostasis and rapidly stopping bleeding, although this may be associated with increased thrombotic risks, all of which will be assessed in this trial.

Observational studies have suggested survival benefits with WB transfusion versus component therapy (mostly at 24 h), with only one study reporting mortality benefit at 30 days (odds ratio 2.19; 95% CI 1.01–4.76; *p* = 0.047) [16, 21, 23]. Three observational studies reported no difference in 30-day mortality (*n* = 830), while one study (*n* = 354) showed a 13% decrease in 30-day mortality with WB transfusion (*p* = 0.002) compared with component therapy [13]. This reported an 8% absolute difference in 24-h mortality between WB (4%) vs component therapy (12%); it also showed a higher proportion of patients receiving massive transfusion in the WB group compared to component therapy (89% vs 78%).

Although there is some evidence of benefit with the use of WB, there have been no prospective randomised trials exploring the clinical and cost effectiveness of pre-hospital administration of WB versus component therapy for bleeding trauma patients. The optimal pre-hospital transfusion strategy has yet to be established and transfusion practice varies across the country. Therefore, it is essential for patients, healthcare professionals, and blood services that the clinical and cost effectiveness of pre-hospital WB transfusion is evaluated in a large trial before its widespread implementation in routine practice.

## Trial status

The current trial protocol is version V1.1 dated 03 May 2022. The trial opened to recruitment on 15 December 2022. Recruitment is anticipated to run for 24 months, and therefore, the estimated completion date is 15 December 2024.

## Data Availability

The final dataset will reside with NHSBT. The chief investigators will have access and can approve exceptional access for other members of the trial team to facilitate analysis or to cover for absences. Access to the final dataset for additional analyses will be permitted under the agreement of the trial review committee (made up of the sponsor representative, chief investigators, trial statistician, and NHSBT information governance) and according to the trial publication policy.

## Abbreviations

AAS: Air Ambulance Service
ACIT-2: Activation of Coagulation and Inflammation in Trauma II
CAG: Confidentiality Advisory Group
CI: Chief investigator
CJD: Creutzfeldt–Jakob disease
CPD: Citrate-phosphate-dextrose
CRF: Case report form
CTU: Clinical Trials Unit
DMC: Data monitoring committee
EQ-5D-5L: EuroQol 5 Dimension 5 Level
EQ-5D-Y: EuroQol 5 Dimension Youth
FFP: Fresh frozen plasma
GCP: Good Clinical Practice
HIV-AIDS: Human immunodeficiency virus-acquired immunodeficiency syndrome
ICNARC: Intensive Care National Audit and Research
NHSBT: NHS Blood and Transplant
PCC: Prothrombin complex concentrate
PROPPR: Pragmatic, Randomized Optimal Platelet and Plasma Ratios
RBCs: Red blood cells
RCT: Randomised controlled trial
SAE: Serious adverse event
SHOT: Serious Hazards of Transfusion
SWiFT: Study of Whole Blood in Frontline Trauma
TARN: Trauma Audit and Research Network
TB: Tuberculosis
TMG: Trial management group
TSC: Trial steering committee
TXA: Tranexamic acid
WB: Whole blood
